# Effect of tobacco outlet density on quit attempts in Korea: a multi-level analysis of the 2015 Korean Community Health Survey

**DOI:** 10.4178/epih.e2021048

**Published:** 2021-08-03

**Authors:** Jaehyung Kong, Sung-il Cho

**Affiliations:** 1 National Tobacco Control Centre, Korea Health Promotion Institute, Seoul, Korea; 2 Department of Public Health Sciences, Graduate School of Public Health and Institute of Health and Environment, Seoul National University, Seoul, Korea

**Keywords:** Tobacco, Tobacco product, Tobacco industry, Smoking cessation, Multilevel analysis

## Abstract

**OBJECTIVES:**

This study aimed to examine whether the regional density of tobacco outlets in Korea was associated with the likelihood of attempting to quit among smokers

**METHODS:**

This study was designed as a secondary data analysis of a cross-sectional study. Data from the 2015 Korean Community Health Survey and tobacco outlet registrations in 17 metropolitan cities and provinces with 254 communities in Korea were used for the analysis. In total, 41,013 current smokers (≥19 years of age) were included. Multi-level logistic regression analysis was conducted to investigate regional differences associated with smokers’ attempts to quit and to evaluate the effects of individual and regional characteristics on quit attempts.

**RESULTS:**

Higher tobacco outlet density was associated with lower odds of attempting to quit. Smokers who resided in districts with the highest tobacco outlet density were 18% less likely to attempt quitting (odds ratio, 0.82; 95% confidence interval, 0.70 to 0.98) than smokers who resided in the regions with the lowest tobacco outlet density (intraclass correlation coefficient, 0.030).

**CONCLUSIONS:**

This study showed that quit attempts were related to community-level factors, such as tobacco outlet density, as well as other individual factors. These findings support the implementation of national policies restricting the number of tobacco outlets within communities or zones and limiting tobacco marketing in tobacco outlets.

## INTRODUCTION

Over 7 million people die worldwide due to smoking every year [1]. In Korea, the smoking prevalence among males is reported to be 38.1% [2]. With nearly four of every 10 males smoking, the socioeconomic costs associated with smoking-related diseases are estimated to be US$6.1 billion [3].

The determinants of smoking and quitting smoking include individual factors and socioeconomic status. However, environmental factors can promote smoking and hinder smoking cessation [4]. The presence of tobacco outlets is an environmental factor that increases tobacco availability. These outlets are a key location for tobacco marketing, where individuals can access and purchase tobacco. In Korea, tobacco retailers sell tobacco products alongside other goods and services. While tobacco cannot be marketed through traditional media such as television, radio, and the Internet, tobacco marketing in tobacco outlets is legally permitted. Anyone may freely enter tobacco outlets, where they are exposed to tobacco advertisements. Despite these risks, tobacco outlets have not been recognized in tobacco control policies as an important factor associated with smoking and smoking cessation. To prevent smoking and create an environment that encourages smoking cessation, it is necessary to consider the effects of tobacco outlets on smokers.

Numerous studies have reported the effects of tobacco outlets on smoking and smoking behaviour. Greater access to tobacco outlets facilitates purchases of tobacco because of the physical proximity to tobacco products and consumers’ increased exposure to tobacco marketing through displays, advertisements, and sales promotions. Tobacco availability increases receptivity to smoking, thereby promoting the frequency of smoking and tobacco purchasing behaviours [5]. Regions with high tobacco retail density can cause price competition among products, which leads to reduced tobacco prices, further encouraging tobacco purchases [6]. Adults who reside in regions with high tobacco outlet densities are reportedly more likely to start smoking than those who live in regions with lower tobacco outlet densities [7]. Moreover, tobacco outlet density hinders the likelihood of successfully quitting smoking. Individuals living in regions with high concentrations of tobacco retailers exhibit low self-efficacy related to smoking, are less likely to consider smoking cessation [8], and are less likely to attempt smoking cessation (odds ratio [OR], 0.54) [9]. Daily smokers have been reported to reside in regions with high tobacco outlet densities and are known to make fewer attempts to quit, compared to those who rarely smoke [10]. Individuals with greater access to tobacco retailers who attempt to quit are reportedly less likely to successfully cease smoking [11-14]. Furthermore, tobacco outlet density within a residential area is associated with increased smoking frequency [15], and individuals who reside closer to tobacco retailers have higher levels of tobacco consumption [16].

Based on these known facts, this study aimed to investigate whether the environment related to tobacco retailers impacts adult smokers’ attempts to quit smoking, for the first time in Korea, using nationwide data on tobacco retailers and current smokers. Based on these results, suggestions are made for the improvement of Korea’s tobacco retailer control policies.

## MATERIALS AND METHODS

### Data source and subjects

In this study, data were collected from 254 communities on adult smokers, the number of tobacco outlets, population, area size, and financial independence rates. Adult smokers’ data (i.e., demographic characteristics and smoking behaviours) were obtained from the 2015 Korean Community Health Survey (KCHS). Data from the Ministry of the Interior and Safety (2015) regarding the occupational information of licensed businesses were used to identify all types (e.g., convenience stores, grocery stores, stationary stores, tobacconist shop, etc.) of currently registered tobacco retailers. Data on the community population and area size were gathered from the Ministry of the Interior and Safety (2016) records of local governments and demographics. Data on the financial independence rate, which was defined as the ratio of the local government’s generated revenue (independent income) to the local government’s total revenue, were collected by Local Finance 365, a public finance data system operated by the Ministry of the Interior and Safety, in 2015. Since 2008, the Korea Disease Control and Prevention Agency (formerly Korea Centers for Disease Control and Prevention) has conducted the annual KCHS among male and female ≥ 19 years of age in 17 metropolitan cities and provinces with 254 communities. A sample of 900 individuals per community was selected by multi-level probability sampling, with respect to the community classification and type of residence [17]. Of 228,558 respondents in 2015, 41,678 were identified as current smokers. Of the current smokers, 665 participants were excluded because of responses of “do not know” or refusal to respond to all survey questions. The remaining 41,013 participants were included in this study.

### Variables

This study aimed to investigate current smokers’ experiences with smoking cessation. A current smoker was defined as an individual who had smoked at least 5 packs of cigarettes (equivalent to 100 cigarettes) in their lifetime and currently smoked, either occasionally or on a daily basis. Participants were considered to have attempted quitting if they responded that they “tried to quit smoking in the past 1 year” to the question, “Have you stopped smoking for at least 1 day (24 hours) in an attempt to quit smoking permanently?” Several individual-level and community-level variables were analysed to assess their effects on attempts to quit.

### Individual-level measures

Individual status variables consisted of demographic characteristics and smoking behaviours. The demographic variables included age, education level, marital status, occupation, and household income. The smoking behaviour variables included smoking frequency, number of cigarettes consumed per day, presence or absence of a health professional’s recommendation to quit smoking, smoking cessation education experience, and anti-smoking campaign exposure.

### Community-level measures

The community-level variables included the financial independence rate, community classification, area size, and tobacco outlet density. The 254 communities were divided into quartiles based on their financial independence rates. A higher financial independence rate was considered indicative of a greater ability of a local government to autonomously manage its finances. In this study, metropolitan cities (*gu*) were classified as metropolis, small-medium cities (*si*) as city, and counties (*gun*) as rural. Communities were categorized into quartiles based on area size (km^2^). Lastly, the tobacco outlet density was defined as the number of tobacco outlets per 1,000 residents in a community.

### Statistical analysis

All obtained data were analysed using Stata version 15.0 (StataCorp., College Station, TX, USA). Descriptive statistics were used to analyse general characteristics according to the variables of individual status and community status. Using MLwiN 2.36, multi-level logistic regression analyses were conducted to investigate the association between the community environment and current smokers’ attempts to quit.

The formula used for the analysis was as follows:

$$\log(\frac{p_{ij}}{1-p_{ij}})\;=\;\gamma 00\;+\;\gamma 01z_{j}\;+\;\gamma 10x_{ij}\;+\;\delta_{0j},\;\delta_{0j}\sim N(0,\;\sigma_{\delta }^{2})$$

Multi-level logistic regression analysis was performed using a history of quit attempts as the dependent variable. For the multi-level analyses, we applied a random-intercept model. Three models were used in this study. The basic model (model 1) included only intercepts to identify significant differences in quit attempts between communities, the level 1 model (model 2) included only individual variables, and the level 2 model (model 3) included both individual and community variables. Intraclass correlation coefficients (ICCs) were calculated for each model to determine how much of the distribution was accounted for by community-level variables.

### Ethics statement

The study protocol was approved by the Institutional Review Board (IRB) of Seoul National University (IRB No. E1802/003-006).

## RESULTS

### General characteristics of the study subjects

Of the 41,013 study participants, 91.0% were males and 9.0% were females. Most participants were 40-49 years of age (25.2%), followed by 50-59 years of age (22.3%), 30-39 years of age (19.5%), 60-69 years of age (12.6%), 19-29 years of age (11.4%), and ≥ 70 years of age (9.0%). Participants’ education levels varied; a highschool education was the most common level (38.2%), followed by a college or university program (36.0%), middle school or below (23.2%), and graduate school or above (2.6%). Most participants (65.9%) had a spouse, while 34.1% did not. Most participants were blue-collar workers (58.9%), followed by white-collar workers (21.6%), unemployed (13.7%), and others (5.8%). The monthly household income of <2.00 million Korean won (KRW; 64.2%), followed by 2.00-3.99 million KRW (32.5%), and ≥4.00 million KRW (3.3%). Of current smokers, 91.0% smoked every day and 9.0% smoked some days; 54.8% smoked at least 11 cigarettes per day and 45.2% smoked 10 cigarettes or fewer per day. Nearly one-third (32.7%) of current smokers had attempted to quit smoking within the past year (Table 1).

### Community characteristics

**Figure 1.**
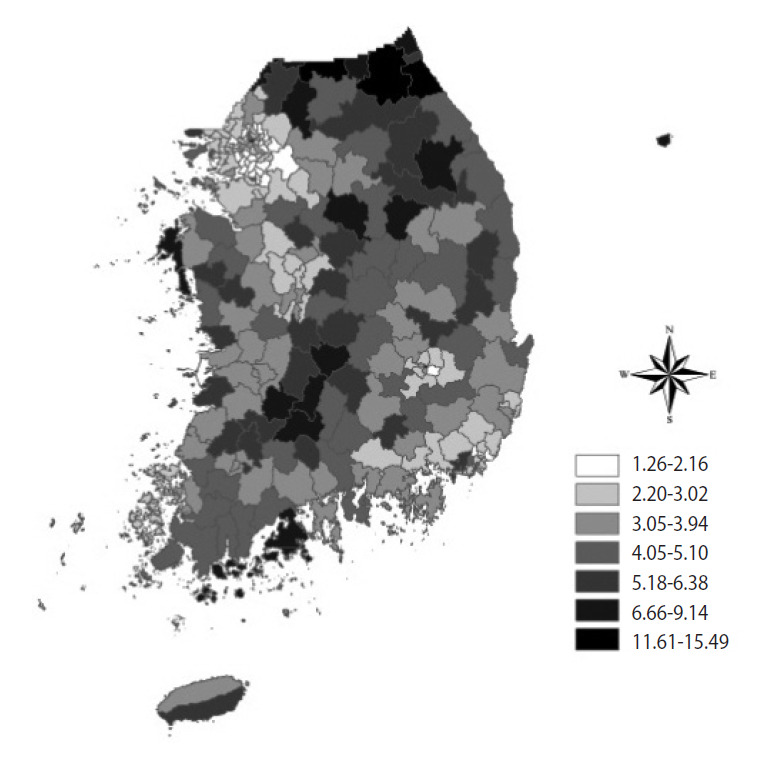
Distribution of tobacco outlet density (per 1,000 persons) across 254 communities in Korea.

The community-level variables, including financial independence rate, area size, and tobacco outlet density, are summarised in Table 2. Among the 254 communities throughout Korea, the mean financial independence rate was 30.2% (range, 9.9 to 64.5). The mean regional area size was 395.0 km^2^ (range, 2.8 to 1,819.8), and the mean tobacco outlet density was 3.9 stores per 1,000 residents (range, 1.3 to 15.5) (Figure 1 and Table 2).

### Effect of tobacco outlet density on quit attempts

Multi-level logistic regression analysis was performed to investigate regional differences associated with smokers’ attempts to quit (i.e., quit attempts), and to evaluate the effects of individual and regional characteristics on these quit attempts. Model 1 was a null model used to investigate the ICC, which indicated how much variation in smokers’ attempts to quit existed between community-level units. Model 2 included individual variables and was therefore used to identify the individual factors that affected quit attempts. In addition to the individual-level characteristics included in model 2, several community-level variables (e.g., financial independence rate, community classification, area size, and tobacco outlet density) were included in model 3. Model 3 was used to investigate whether community-level variables affected individual smokers’ attempts to quit.

The multi-level logistic regression results are summarised in Table 3. Regional differences were found to have statistically significant effects on quit attempts, demonstrating the presence of a local environment effect on smoking cessation. Several individual-level factors were also significantly associated with attempting to quit; these included sex, age, education level, marital status, occupation, smoking frequency, average smoking volume per day, previous experience with smoking cessation education, and a health professional’s recommendation to quit smoking. Of the community-level variables, the financial independence rate, community classification, and area size did not have a significant effect, whereas tobacco outlet density did. Participants who resided in communities in the fourth quartile of tobacco outlet density (i.e., with the highest density) had an OR of 0.82 for attempting to quit, compared to those living in regions in the first quartile of tobacco outlet density (i.e., lowest density).

ICCs were calculated to assess the random effects of each model. The ICC of the basic model (model 1) was 0.037. The community-level variables contributed to 3.7% of the quit attempt distribution. Models 2 and 3 had ICCs of 0.031 and 0.030, respectively. The community-level variables had significant effects on quit attempts, despite smaller contributions to the overall distribution.

## DISCUSSION

Multi-level analyses were carried out to identify individual-level and community-level factors affecting adult smokers’ quit attempts. The results showed that individual-level factors with significance include sex, age, education level, marital status, occupation, smoking frequency, daily average smoking amount, previous experience with smoking cessation education, and a health professional’s recommendation to quit smoking. In terms of community-level factors, the density of tobacco retailers in the community had significance, even after controlling for individual-level factors.

Such results were similar to those of previous studies that quit smoking rate is lower in females than in males [18] and that strategies to promote the intention to quit smoking are required as 47.8% of female smokers have no intention to quit [19]. The results are also consistent with the outcome of Ahn [20]’s research that the likelihood of quit attempts decreased with age. Other than the physical difference between males and females, this tendency seems to come from the fact that female smokers do not receive proper smoking cessation education or advice because they are not able to openly express their smoking problems or intention to quit smoking due to social norms. The finding regarding age, the outcome seems to reflect smokers’ view that quitting their long-term habit of smoking in old age would not practically be effective for health [21]. Smokers with spouses were much more likely to attempt to quit smoking than those without spouses, which coincides with the results of Abdullah et al. [22]. As was found by Ryu et al. [23] and Jeon [24], higher education levels and lower smoking amounts were associated with a higher likelihood of attempting to quit. Moreover, the likelihood of quit attempts was higher in smokers who had experienced smoking cessation education [20,23] and those who had received recommendations to quit smoking from health professionals [25], aligning with previous research.

This study also found that there were fewer attempts to quit among smokers living in communities with high tobacco outlet densities than among smokers living in communities with low tobacco outlet densities. The findings are consistent with the results of research conducted in Scotland, which showed that the likelihood of quitting smoking was lower among smokers living in regions where tobacco retailers are highly concentrated [13]. This is also consistent with previous reports that smokers in such regions had low self-efficacy related to smoking and were unlikely to consider quitting smoking [8].

Tobacco outlets may affect smokers and smoking behaviours in several ways. Visible tobacco products and advertising increase the likelihood of smoking initiation, enhance brand awareness, and facilitate unplanned purchases. Tobacco outlets both provide routes through which smokers can purchase tobacco and expose smokers to tobacco advertisements and promotions. A higher regional tobacco outlet density reduces the cost and effort for smokers to purchase tobacco; these changes lead to increased tobacco purchasing behaviour [5,11] and reduced smoking cessation intent. Furthermore, because tobacco outlets directly expose consumers to advertisements and promotions, smokers are more frequently exposed to tobacco advertisements and sales promotions when there are more tobacco outlets in the region. Previous studies have shown that smokers exposed to tobacco advertisements and sales promotions exhibit an increased desire to smoke [26] and an increased likelihood to purchase tobacco impulsively [27], both of which hinder their ability to quit smoking.

Given the effects of tobacco outlets on smokers and community members, these outlets should be considered in tobacco control policies. Particularly in Korea, unlike other countries, retailers selling other goods or services such as convenience stores, supermarkets, lottery shops, stationery stores, and hardware stores sell and advertise tobacco as well. This is the reason for the existence of so many tobacco retailers in our daily lives, which also leads to a higher retailer-to-population ratio in cities and rural areas than in metropolises. Thus, policies to control tobacco retailers are necessary to close the gap in health disparities due to smoking. Currently, Korea has lenient licensing criteria for tobacco outlets and minimal restrictions regarding the number of tobacco retailers within specific regions. In addition, there are insufficient regulations on tobacco advertisements and promotions within tobacco outlets.

There are several challenges involved in the implementation of effective tobacco control policy. Since tobacco retailers are licensed and managed by local governments under the Tobacco Business Act, the development and implementation of integrative management of tobacco retailers at the regional level may prove challenging. In addition, it is difficult to make generalizations regarding the tobacco retailer distribution process among regions. Furthermore, the law does not restrict the number of tobacco retailers within a region—it only stipulates that outlets must be at least 50 meters apart from each other; therefore, new retailers continue to emerge. In Korea, tobacco advertisements can be legally displayed inside outlets, allowing tobacco companies to post a variety of enticing advertisements inside these outlets.

Some countries have implemented policies to restrict the locations and numbers of tobacco retail stores. There is also a policy to restrict tobacco sales and advertising stores through local community initiatives, and discussions are being held to monitor compliance and strengthen public awareness [28].

This study has some limitations that should be addressed. First, since the research data did not include individual smokers’ home addresses, tobacco outlet density had to be defined as the number of tobacco retailers per population. More accurate results could have been obtained if areas or routes frequently used by respondents could have been investigated based on information about individual smokers’ addresses. Second, only a few community level factors were considered. Few previous studies have investigated community-level factors affecting smoking cessation, and a broader range of factors should be considered. Third, this study has the inherent limitation of all cross-sectional studies: since the research data were obtained only at a specific point in time, and not over time, they do not provide information regarding changes in the variables or causal relationships between the variables. In addition, further studies should be conducted considering the possibility that the tobacco industry may have established more tobacco retailers in the areas where the smoking population and tobacco consumption are high to promote tobacco marketing.

Despite these limitations, this study is meaningful in that it used nationwide data about individual and community status, surveyed at the community-level, to investigate the overall distribution of tobacco retailers across the country, the characteristics of current smokers, and the factors related to individual and community status that affect quit attempts by smokers. Furthermore, based on the knowledge that a higher density of tobacco outlets reduces the possibility of quitting smoking, it was confirmed that the tobacco outlet density in the community affects the likelihood of attempting to quit among smokers. This means that a community-level strategy for tobacco outlet control is needed to cope with this risk factor for community health.

This study identified a need for emphasizing tobacco supply reduction measures. Efforts to reduce tobacco use may include restrictions on the locations and numbers of tobacco outlets, while considering regional characteristics. Furthermore, regulations on tobacco marketing (e.g., tobacco displays, advertisements, and promotions) within tobacco outlets should be considered to create environments conducive to smoking cessation.

## Figures and Tables

**Table 1.** General characteristics of the study subjects

Characteristics	n (%)
Current smoker (n=41,013)	
Demographic status	
Sex	
Male	37,313 (91.0)
Female	3,700 (9.0)
Age (yr)	
19-29	4,653 (11.4)
30-39	7,999 (19.5)
40-49	10,346 (25.2)
50-59	9,145 (22.3)
60-69	5,190 (12.6)
≥70	3,680 (9.0)
Education	
Middle school or less	9,503 (23.2)
High school	15,682 (38.2)
College/university	14,783 (36.0)
Graduate school or more	1,045 (2.6)
Spouse	
No	13,995 (34.1)
Yes	27,018 (65.9)
Occupation	
Unemployed	5,615 (13.7)
Blue-collar worker	24,173 (58.9)
White-collar worker	8,853 (21.6)
Other	2,372 (5.8)
Household income (10^4^ Korean won)	
<200	26,337 (64.2)
200-399	13,344 (32.5)
≥400	1,332 (3.3)
Smoking status	
Smoking frequency	
Occasional	3,696 (9.0)
Daily	37,317 (91.0)
Quit attempt	
No	27,580 (67.3)
Yes	13,433 (32.7)
No. of cigarettes per day	
≤10	18,544 (45.2)
≥11	22,469 (54.8)
Experience of smoking cessation education	
No	36,885 (89.9)
Yes	4,128 (10.1)
Recommendation from a health professional to quit smoking	
No	28,073 (68.5)
Yes	12,940 (31.5)
Exposure to anti-smoking campaigns	
No	4,722 (11.5)
Yes	36,291 (88.5)

**Table 2.** Descriptive statistics for community-level variables

Variables	Mean	Standard deviation	Minimum	Maximum
Financial independence rate (%)	30.2	13.3	9.9	64.5
Area size (km^2^)	395.0	369.2	2.8	1,819.8
Tobacco outlet density (n)	3.9	1.9	1.3	15.5
Population	202,871.4	161,939.8	10,153.0	660,302.0
Tobacco outlets	607.9	387.1	70.0	2,085.0

**Table 3.** Multi-level analysis result of factors associated with smokers’ quit attempts

Variables	Smokers’ quit attempts	
	Model 2	Model 3
Fixed effects		
Individual-level		
Sex		
Male	1.00 (reference)	1.00 (reference)
Female	0.81 (0.74, 0.88)^***^	0.81 (0.74, 0.88)^***^
Age (yr)		
19-29	1.00 (reference)	1.00 (reference)
30-39	0.82 (0.76, 0.90)^***^	0.83 (0.76, 0.91)^***^
40-49	0.69 (0.63, 0.75)^***^	0.69 (0.63, 0.76)^***^
50-59	0.65 (0.59, 0.71)^***^	0.65 (0.59, 0.72)^***^
60-69	0.63 (0.56, 0.71)^***^	0.64 (0.57, 0.71)^***^
≥70	0.47 (0.41, 0.54)^***^	0.48 (0.42, 0.55)^***^
Education		
Middle school or less	1.00 (reference)	1.00 (reference)
High school	1.10 (1.02, 1.18)^**^	1.10 (1.02, 1.18)^**^
College/university	1.18 (1.09, 1.28)^***^	1.18 (1.09, 1.29)^***^
Graduate school or more	1.33 (1.13, 1.55)^***^	1.32 (1.12, 1.53)^**^
Spouse		
No	1.00 (reference)	1.00 (reference)
Yes	1.13 (1.07, 1.19)^***^	1.13 (1.08, 1.19)^***^
Occupation		
Unemployed	1.00 (reference)	1.00 (reference)
Blue-collar	1.14 (1.06, 1.24)^**^	1.15 (1.06, 1.25)^**^
White-collar	1.18 (1.08, 1.29)^**^	1.18 (1.08, 1.30)^***^
Other	1.19 (1.06, 1.34)^**^	1.20 (1.06, 1.36)^**^
Household income (10^4^ Korean won)		
<200	1.00 (reference)	1.00 (reference)
200-399	0.97 (0.92, 1.02)	0.96 (0.91, 1.01)
≥400	1.10 (0.97, 1.24)	1.09 (0.97, 1.24)
Smoking frequency		
Occasional	1.00 (reference)	1.00 (reference)
Daily	0.34 (0.31, 0.36)^***^	0.34 (0.31, 0.37)^***^
No. of cigarettes per day		
≤10	1.00 (reference)	1.00 (reference)
≥11	0.66 (0.63, 0.69)^***^	0.66 (0.63, 0.69)^***^
Experience of smoking cessation education		
No	1.00 (reference)	1.00 (reference)
Yes	1.55 (1.44, 1.67)^***^	1.56 (1.45, 1.67)^***^
Recommendation from a health professional to quit smoking		
No	1.00 (reference)	1.00 (reference)
Yes	1.15 (1.10, 1.21)^***^	1.15 (1.10, 1.21)^***^
Exposure to anti-smoking campaigns		
No	1.00 (reference)	1.00 (reference)
Yes	1.04 (0.96, 1.11)	1.04 (0.96, 1.11)
Community-level		
Financial independence rate (%)^1^		
1st quartile (≤19.6)	-	1.00 (reference)
2nd quartile (≤25.8)	-	1.10 (0.98, 1.25)
3rd quartile (≤41.5)	-	1.02 (0.90, 1.16)
4th quartile (>41.5)	-	1.11 (0.96, 1.28)
Community classification		
Rural	-	1.00 (reference)
City	-	1.07 (0.90, 1.26)
Metropolis	-	1.08 (0.83, 1.29)
Area size (km^2^)^1^		
1st quartile (≤47.2)	-	1.00 (reference)
2nd quartile (≤378.3)	-	1.13 (0.99, 1.28)
3rd quartile (≤631.9)	-	1.10 (0.91, 1.29)
4th quartile (>631.9)	-	1.08 (0.89, 1.30)
Tobacco outlet density (per 1,000 persons)^1^		
1st quartile (≤2.7)	-	1.00 (reference)
2nd quartile (≤3.5)	-	0.93 (0.80, 1.07)
3rd quartile (≤4.7)	-	0.95 (0.83, 1.11)
4th quartile (>4.7)	-	0.82 (0.70, 0.98)^*^
Random effect		
Variance of community (SD)	0.104 (0.013)	0.100 (0.012)
ICC	0.031	0.030

Model 2, adjusted for individual level variables; Model 3, adjusted for individual and community level variables. OR, odds ratio; CI, confidence interval; KRW, Korean won; SD, standard deviation; ICC, intraclass correlation coefficient

1 For all variables measured using quartiles, the first quartile is the lowest and the fourth quartile is the highest.

* p<0.05,

** p<0.01,

*** p<0.001.
